# Using Twitter Data to Gain Insights into E-cigarette Marketing and Locations of Use: An Infoveillance Study

**DOI:** 10.2196/jmir.4466

**Published:** 2015-11-06

**Authors:** Annice E Kim, Timothy Hopper, Sean Simpson, James Nonnemaker, Alicea J Lieberman, Heather Hansen, Jamie Guillory, Lauren Porter

**Affiliations:** ^1^ RTI International Research Triangle Park, NC United States; ^2^ Distil Networks (at RTI International at the time of the study) Raleigh, NC United States; ^3^ Rady School of Management University of San Diego San Diego, CA United States; ^4^ Bureau of Tobacco Free Florida Florida Department of Health Tallahassee, FL United States

**Keywords:** electronic cigarettes, social media, tobacco, marketing, natural language processing

## Abstract

**Background:**

Marketing and use of electronic cigarettes (e-cigarettes) and other electronic nicotine delivery devices have increased exponentially in recent years fueled, in part, by marketing and word-of-mouth communications via social media platforms, such as Twitter.

**Objective:**

This study examines Twitter posts about e-cigarettes between 2008 and 2013 to gain insights into (1) marketing trends for selling and promoting e-cigarettes and (2) locations where people use e-cigarettes.

**Methods:**

We used keywords to gather tweets about e-cigarettes between July 1, 2008 and February 28, 2013. A randomly selected subset of tweets was manually coded as advertising (eg, marketing, advertising, sales, promotion) or nonadvertising (eg, individual users, consumers), and classification algorithms were trained to code the remaining data into these 2 categories. A combination of manual coding and natural language processing methods was used to indicate locations where people used e-cigarettes. Additional metadata were used to generate insights about users who tweeted most frequently about e-cigarettes.

**Results:**

We identified approximately 1.7 million tweets about e-cigarettes between 2008 and 2013, with the majority of these tweets being advertising (93.43%, 1,559,508/1,669,123). Tweets about e-cigarettes increased more than tenfold between 2009 and 2010, suggesting a rapid increase in the popularity of e-cigarettes and marketing efforts. The Twitter handles tweeting most frequently about e-cigarettes were a mixture of e-cigarette brands, affiliate marketers, and resellers of e-cigarette products. Of the 471 e-cigarette tweets mentioning a specific place, most mentioned e-cigarette use in class (39.1%, 184/471) followed by home/room/bed (12.5%, 59/471), school (12.1%, 57/471), in public (8.7%, 41/471), the bathroom (5.7%, 27/471), and at work (4.5%, 21/471).

**Conclusions:**

Twitter is being used to promote e-cigarettes by different types of entities and the online marketplace is more diverse than offline product offerings and advertising strategies. E-cigarettes are also being used in public places, such as schools, underscoring the need for education and enforcement of policies banning e-cigarette use in public places. Twitter data can provide new insights on e-cigarettes to help inform future research, regulations, surveillance, and enforcement efforts.

## Introduction

Electronic cigarettes (e-cigarettes) have grown in popularity since their introduction into the marketplace in 2006. E-cigarette awareness is high among adults in the United States (73%) and abroad [1-3]. The proportion of US adults who have ever used e-cigarettes increased rapidly from 1.8% in 2010 to 10.0% in 2013, with use rates highest among young adults and current cigarette smokers [4]. Today, more US teens use e-cigarettes than traditional cigarettes [5-7]; nationally, 9% of 8th graders, 16% of 10th graders, and 17% of 12th graders reported e-cigarette use in the past 30 days compared with 4% of 8th, 7% of 10th, and 14% of 12th graders reporting cigarette use in the past 30 days [5].

Consumer interest in and use of e-cigarettes may be influenced by advertising and information sharing from social sources. E-cigarette advertising expenditures increased dramatically across media channels between 2010 and 2013, including television, magazines, outdoor, radio, and online [8-10]. E-cigarette television ads increased by 256% from 2011 to 2013 [9] and more than US $2 million was spent on e-cigarette and tobacco ads online from 2012 to 2013 [10]. Adults and youth are receptive to e-cigarette television ads [8,11] and exposure is associated with intentions to use e-cigarettes among youth [11]. However, studies show that most people heard about e-cigarettes online (41%) or from personal contacts (35%) [12] and that most consumers who try e-cigarettes do so out of curiosity or because a friend or family member offered it to them [13]. Recent studies have documented that e-cigarette information is widely available online from branded websites, e-cigarette user forums, marketing, and user-generated content on social media sites such as Twitter and YouTube [14,10]. Therefore, understanding how e-cigarettes are marketed and what information consumers share about them could help to inform ongoing surveillance and regulatory efforts.

Social media is an important source of information in our everyday lives. In the United States, nearly 81% of youth [15] and 74% of adults [16,17] use some form of social media. Twitter, a social networking microblog, has grown in popularity and currently (September 17, 2015) has more than 316 million active users [18]. Registered users can publish an unlimited number of 140-character posts (“tweets”) that are by default visible to the public. As of September 2015, users are creating more than 500 million tweets daily [18]. Because tweets are publicly available, Twitter has become a rich data source for surveillance of public health issues and insights into emerging phenomena [19-26].

Researchers have begun to explore e-cigarette conversations on Twitter [27-29]. Huang and colleagues [29] analyzed e-cigarette tweets from May to June 2012 and found that 90% of tweets were commercial/advertising-related and that these tweets emanated from a relatively small subset of extremely active users. Also, most of these tweets (94%) included URLs, which in many cases were used for the promotion or sale of e-cigarettes. In another study, researchers found that Twitter was used to oppose passage of e-cigarette regulation [28]. The week before the Chicago City Council was scheduled to vote on regulating e-cigarettes as a tobacco product, most tweets mentioning the Chicago Department of Public Health (59%) were against the policy and framed e-cigarettes as healthier alternatives to cigarettes and as aids to smoking cessation. Findings from this study suggest that 14% of these tweets were created using accounts intended to create perceptions of consensus regarding an issue (eg, health benefits of e-cigarettes). These studies suggest that Twitter data may be useful for surveillance of e-cigarette marketing and policy issues.

E-cigarettes are currently not regulated by the US Food and Drug Administration (FDA), but the FDA has issued a deeming rule to extend their authority to regulate e-cigarettes. Researchers have argued that swift responses to e-cigarette advertising are needed [30]. If the FDA regulates the manner e-cigarettes are marketed, including the type of claims companies can make, ongoing surveillance of e-cigarette marketing practices and enforcement of potential violators is needed. Because e-cigarettes are advertised online, identifying and monitoring these entities will be critical to regulatory enforcement efforts, especially on platforms such as social media where multiple accounts can be opened with no verification of one’s true identity. Therefore, identifying marketers who are advertising e-cigarettes online will be critical to these efforts.

There has been more active local and state government regulations including banning sales of e-cigarettes to minors [31] and prohibiting use of e-cigarettes in public places by amending clean indoor air laws to include e-cigarettes. However, the extent to which these laws are enforced is largely unknown. In an online survey of 1201 adult US adult smokers, we found that 35.0% reported seeing others use e-cigarettes “often” or “very often” in public places, such as restaurants (31.6%), parks and beaches (43.7%), and worksites (21.0%) [32]. Because social media is widely used by consumers, analyzing social media data may provide more insights into consumer behaviors and reactions to e-cigarette policies that could help inform future regulatory action.

Our study explored insights about e-cigarettes gleaned from mining Twitter data across multiple years (2008-2013) from the Twitter firehose (full sample of tweets). This study builds on previous research studying e-cigarette marketing on Twitter in several important ways [29]. First, our Twitter data and analyses represent a crucial period (2008-2013) for understanding rapid increases in e-cigarette marketing, advertising, sales, and use. These analyses capture tweets during the period when e-cigarette use began to increase in popularity. Second, this study identifies marketing trends that may be indicative of spamming or fake consumers (eg, purchasing followers), which has important implications for prioritizing regulatory efforts for e-cigarette marketing. Third, this study derives the places where people report using (and seeing others use) e-cigarettes from tweets, which helps to illuminate locations where policy makers should consider passing regulations to prohibit the use of e-cigarettes.

## Methods

### Data Source

Twitter data were obtained from Radian6 [33], a leading social media monitoring tool that collects data from more than 400 million sources across social networking sites (eg, Twitter, Facebook, YouTube), forums, blogs, and mainstream news. Radian6 offers historical Twitter data from July 1, 2008, and provides users full access to all available tweets from the Twitter firehose.

### Keyword Search

To identify tweets about e-cigarettes, we developed a search syntax that included 55 search keywords, including general e-cigarette terms (eg, electronic cigarette, eCig), specific e-cigarette brand names (eg, blu, NJoy, green smoke), and terms about e-cigarette use (eg, vaping). We reviewed the initial search results and amended the syntax to exclude other tobacco or drug terms (eg, marijuana, hookah). The final search syntax was entered into Radian6 to identify relevant tweets from July 1, 2008 to February 28, 2013 (when the search was conducted). The search results were downloaded into Microsoft Excel from Radian6, including the date and time of the tweet, Twitter handle, the entire text of the tweet (including URLs, hashtags), and the number of followers at the time the tweet was posted.

### Tweet Classification and Analysis

Data processing was done in the Python programming languages. We extracted hashtags and links from the text using regular expressions [34]. For hashtags, we used the regular expression #[a-zA-Z][a-z-A-Z0-9_]. For URLs, we used +:/{2}[-]+(.[-]+)(?:(?:/[^s/])) [34]. Because many links are URL shorteners (eg, bit.ly, t.co), we unshortened the URLs using the application programming interfaces (APIs) provided by unshort.me and Unshorten.It! [35].

### Classification of Advertising Tweets

We randomly selected 507 tweets from the Radian6 corpus and manually classified them as advertising or not advertising. A tweet was coded as advertising e-cigarettes if it mentioned specific brands or websites and listed a price, promotional offer, and words such as “buy.” By using the scikit-learn module in Python [36], we built a classification algorithm to tag the remaining tweets as advertising or not. We used text content of the tweet and 5 metadata features in our classification algorithm: follower count at time of tweet, following count at time of tweet, number of tweets at time of tweet, a binary feature indicating whether or not the tweet contained a URL, and a binary feature indicating whether or not the tweet was a retweet.

To turn the tweet text into a feature matrix [37], we used scikit-learn’s CountVectorizer method [36]. CountVectorizer creates a feature for each word and, in this case, a feature for every n-gram of length 1 to 4 [37]. For each tweet, the value of the feature for a given n-gram was one if the tweet contained the n-gram and zero otherwise. Initially, we had 15,313 n-gram features along with the 5 metadata features.

Because the number of features was 30 times the number of data points, we performed feature selection before training a classification model [38,39]. We trained an extremely randomized trees model [39] (implemented in scikit-learn as ExtraTreesClassifier) on our data and selected only the features with nonzero feature importance. This resulted in 2167 features. We then fit the reduced data with a random forest model (as implemented by scikit-learn) with 10 trees. We scored our model with a tenfold cross-validation according to the accuracy metric (proportion correctly classified). (Because we were concerned about correct classification in both classes, precision and recall were insufficient metrics here.) The mean accuracy across the 10 cross-validations was 0.907. Because 78% of the tweets in the training data were advertising, the naive classifier that labeled everything as advertising would result in an accuracy of 0.78. Our model was a 16% improvement over this baseline. The classifier had high precision (91%) and recall (93%).

### Classification of Where People Use E-Cigarettes

By using a combination of natural language processing and manual classification, we extracted prepositional phrases indicating where people used e-cigarettes or observed others using them. We started with the corpus of tweets that we classified as not advertising.

First, we selected a subset of the tweets containing the words *smoking*, *vaping*, *smokes*, *vapes*, *smoked*, and *vaped* that indicated active usage of e-cigarettes. Second, we cleaned each of these tweets according to a series of rules that were iteratively developed to maximize the number of prepositional phrases extracted. These rules included removing @-mentions and hashtags, removing various interjections (eg, “lol,” “smh,” and various expletives), removing common subordinate prepositional phrases (eg, “in the middle,” “in front”), and correcting common misspellings and slang commonly used in text-based communication (eg, “ur” to “you’re”). Third, we ran each cleaned tweet through the parts-of-speech tagger in the Natural Language Toolkit (NLTK) library [40]. The tagger attempted to label each word in a document (ie, tweet) with its part of speech. Fourth, to extract prepositional phrases from the tweets, we extracted each preposition and all subsequent words before the next verb, preposition, or “wh-” adverb (eg, “whenever,” “where”). Statistical methods for extraction of prepositional phrases, such as the Stanford Parser [41] are available. However, because of the prevalence of poor spelling and bad grammar on Twitter, the Stanford Parser proved ineffective at correctly extracting prepositional phrases. Looking at a sample of 100 tweets containing prepositional phrases, we determined that the 3 primary prepositions indicating the location of e-cigarette use were “in” (62 tweets), “at” (12 tweets), and “on” (11 tweets). The next most frequent prepositions were “around” (3 tweets) and “during” (3 tweets). Thus, we restricted further analysis to tweets containing one of the top 3 prepositions. Finally, we manually compiled a list of nouns that commonly occur in prepositional phrases, but do not refer to a physical location (eg, at “night,” in “life”). Prepositional phrases containing these words as objects were excluded from our analysis. Once prepositional phrases with these objects were excluded, we were left with a list of tweets containing references to e-cigarettes being used in physical locations.

## Results

**Figure 1:**
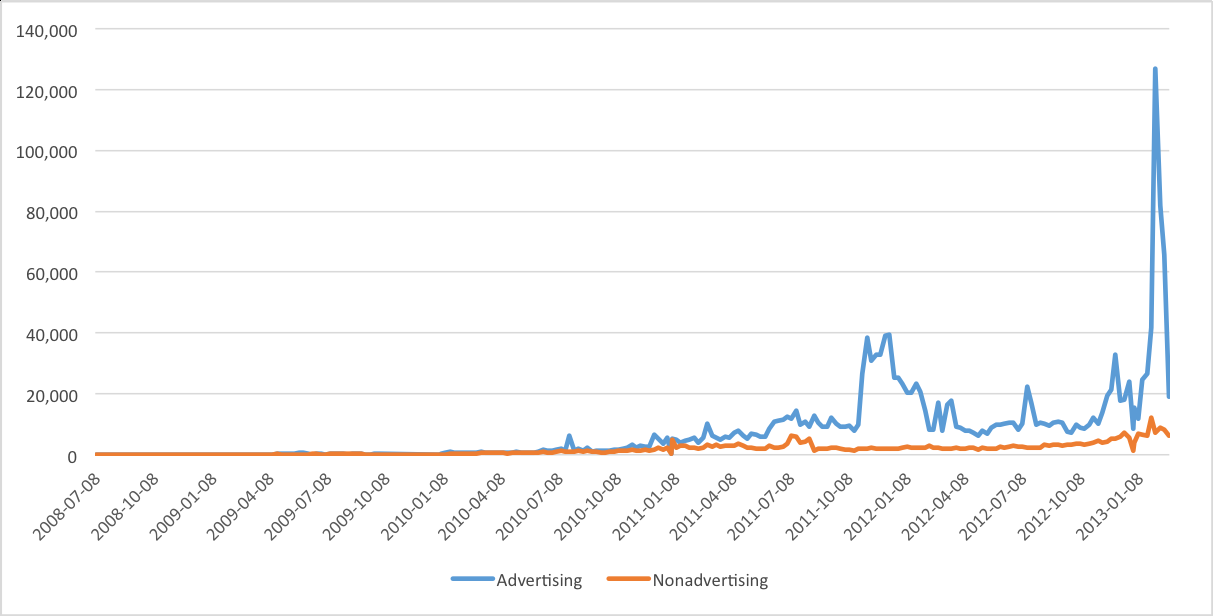
Number of e-cigarette tweets by type (advertising vs nonadvertising), weekly from July 1, 2008 to February 28, 2013.

We identified a total of 1,669,123 tweets about e-cigarettes from July 1, 2008 to February 28, 2013 (Figure 1). The number of e-cigarette tweets increased from 82 in 2008 (May through December only) to 10,870 in 2009, 141,405 in 2010, 746,541 in 2011, 643,900 in 2012, and 64,734 in 2013 (January and February only) (Table 1). Of these 1.6 million tweets, 93.43% (n=1,559,508) were advertising e-cigarettes, whereas only 6.57% (n=109,615) were nonadvertising tweets (Table 1). Approximately 28.70% (447,579/1,559,508) of advertising tweets were retweeted, whereas 11.60% (12,715/109,615) of nonadvertising tweets were retweeted. As Figure 1 shows, advertising tweets increased dramatically over the period observed with periods of sharp increases and declines, whereas nonadvertising tweets increased minimally and at a relatively stable rate.

We are unaware of any events that could explain the spikes observed. We examined the spike around October/November 2011 and noticed an increase in the number of Twitter handles that started posting e-cigarette content at that time but stopped in early 2012. We think this may have been a coordinated marketing/spam effort because many of the Twitter accounts were random numbers and characters.

Table 1 summarizes characteristics of advertising and nonadvertising tweets. Approximately 10% of the advertising tweets described price-related promotions, including coupons (7.69%, 119,904/1,559,508), percent off (7.61%, 118,616/1,559,508), and discount offers (0.89%, 13,952/1,559,508). Approximately 14.99% (233,712/1,559,508) mentioned one of the 32 e-cigarette brands we coded for, with blu (5.99%, 93,405/1,559,508), V2 (2.05%, 31,983/1,559,508), and Green Smoke (1.78%, 27,778/1,559,508) being the most commonly mentioned brands. The 5 most active Twitter handles over the study period produced between 32,141 and 88,424 tweets. Two of the 5 handles included words related to e-cigarettes (eg, vapor and e-cigarettes). The most prolific handle belonged to an online e-cigarette vendor with the following Twitter profile: “Over 125 flavors of Ejuice to choose from at [Vapor God website]. We also offer a Flavor Lab where you can create your own ejuice flavors!” As of March 7, 2015, this handle had 15,601 followers and had posted 107,739 tweets, but only 10 tweets were posted since November 5, 2012. Many of their tweets were of the following form, with different flavors advertised: “Try our great-tasting vanilla cupcake flavored ecigarette eliquid! Get 20% off at checkout with coupon code-twitter [Vapor God website] (Nov 5, 2012).” In contrast, the other active Twitter handles had limited information on their profile (ie, no description, custom background, picture, or URL), and the name given for the account was generic (eg, “moou,” “alejandro”). These Twitter handles also had few followers (132-1340) compared with the most prolific Twitter handle, yet they had a large number of total tweets (eg, one account posted 132,242 tweets as of March 7, 2015). Most tweets were short fragments of text promoting e-cigarettes (eg, “Review e-cigarette” and “Best vapor 7.5mm ecigs”) often with a link that redirected to an inactive page at the time of last review (March 7, 2015). Tweets also contained e-cigarette promotional phrases within nonsensical strings of words (eg, “Buy Electronic smoke The e cigarette bass viol safeguarding brace the healthiness but the...e-Cigarettes On Sale”). This pattern suggests that an automated computer program, rather than a human, may have been generating tweet content and posting it online. None of these Twitter handles appeared to be active as of December 2013 and one account was suspended by Twitter. The most common links shared in advertising tweets are summarized in Table 1. The link to the VaporGod website [42] was shared in 89,068 tweets, mostly by the most prolific Twitter handle for e-cigarette advertising tweets. The top 3 links most commonly shared by unique Twitter handles [43-45] appeared to be affiliate sites with news and reviews about e-cigarettes, including advertisements for e-cigarette brands and links to free e-cigarette starter kits.

Characteristics of the 109,615 nonadvertising e-cigarette tweets are also summarized in Table 1. Specific brands were mentioned in 4244 nonadvertising tweets with top mentions being blu (979 tweets, 0.89%), Vapor4life (803 tweets, 0.73%), Volcano (718 tweets, 0.66%), NicStick (605 tweets, 0.55%), and eSmoke (311 tweets, 0.28%). The most active Twitter handles among the nonadvertising tweets produced between 424 and 1224 tweets. All these Twitter handles included e-cigarette–related terms in the handle (eg, vape, ecigs). The nonadvertising Twitter handles had substantially fewer followers (51-2705) and tweeted less content than the top e-cigarette advertising Twitter handles. According to its Twitter profile, the most prolific nonadvertising Twitter handle is a “global electronic cigarette manufacturer & retailer,” but this retailer is tweeting about e-cigarette–related policies and news stories and interacting with followers, rather than advertising its products. Another profile notes that he is a “husband, father of 4, nurse and vaper” who “Love[s] technology and getting outside when I can. Spending time with my kids & fishing are my favorite things to do.”

**Table 1:** Number and characteristics of e-cigarette tweets, May 1, 2008 to February 28, 2013 (N=1,669,123).

Characteristic			Tweets
**Number of tweets, n**			
	2008 (July-December only)		82
	2009		10,870
	2010		141,405
	2011		746,541
	2012		643,900
	2013 (January-February only)		64,734
	**Total, n (%)**		1,669,123 (100)
		Advertising tweets	1,559,508 (93.43)
		Nonadvertising tweets	109,615 (6.57)
**Advertising tweets, n (%)**			
	Promotion		152,812 (9.80)
	Coupon		119,904 (7.69)
	Percent Off		118,616 (7.61)
	Discount		13,952 (0.89)
	**Brands mentioned**		233,712 (14.99)
		blu	93,405 (5.99)
		V2	31,983 (2.05)
		Green Smoke	27,778 (1.78)
		Premium	11,112 (0.71)
		Luci	9337 (0.60)
	**Most active Twitter handles** ^a^		
		Most active handle (16,160 followers)	88,424 (5.67)
		Second most active handle (1393 followers)	50,651 (3.25)
		Third most active handle (136 followers)	41,032 (2.63)
		Fourth most active handle (145 followers)	36,694 (2.35)
		Fifth most active handle (149 followers)	32,141 (2.06)
	**Most common links shared in tweets**		
		VaporGod [42]	89,068 (5.71)
		http://aan.atrinsic.com/z/873949/9092/&subid1=9546	75,580 (4.85)
		http://bestcelebrex.blogspot.com/p/e-cigarette.html	42,751 (2.74)
		South Beach Smoke	14,718 (0.94)
		http://ECigarettesStarterKits.com [43]	8351 (0.54)
**Nonadvertising tweets, n (%)**			
	**Brands mentioned**		4244 (3.87)
		blu	979 (0.89)
		Vapor4Life	803 (0.73)
		Volcano	718 (0.66)
		NicStick	605 (0.55)
		eSmoke	311 (0.28)
	**Most active Twitter handles**		
		Most active handle (2705 followers)	1224 (1.11)
		Second most active handle (646 followers)	899 (0.82)
		Third most active handle (702 followers)	737 (0.67)
		Fourth most active handle (51 followers)	547 (0.50)
		Fifth most active handle (246 followers)	424 (0.39)
	**Places mentioned in tweets**		471 (0.43)
		Class	184 (39.07)
		House/room/in bed	59 (12.53)
		School	57 (12.11)
		Public place	41 (8.70)
		Bathroom	27 (5.73)
		Work	21 (4.46)
		In front of someone	12 (2.55)
		Car	11 (2.34)
		Restaurant	10 (2.1)
		Movie theater	9 (1.91)
		Airplanes/airport	8 (1.70)
		Store	7 (1.49)
		Bars/clubs	6 (1.27)
		Dormitory	6 (1.27)
		Library	4 (0.85)
		Mall	3 (0.64)
		Bowling alley	2 (0.42)
		Café/coffee shop	2 (0.42)
		Hospital	1 (0.21)
		Locker room	1 (0.21)

^a^Number of followers as of December 12, 2013.

**Figure 2:**
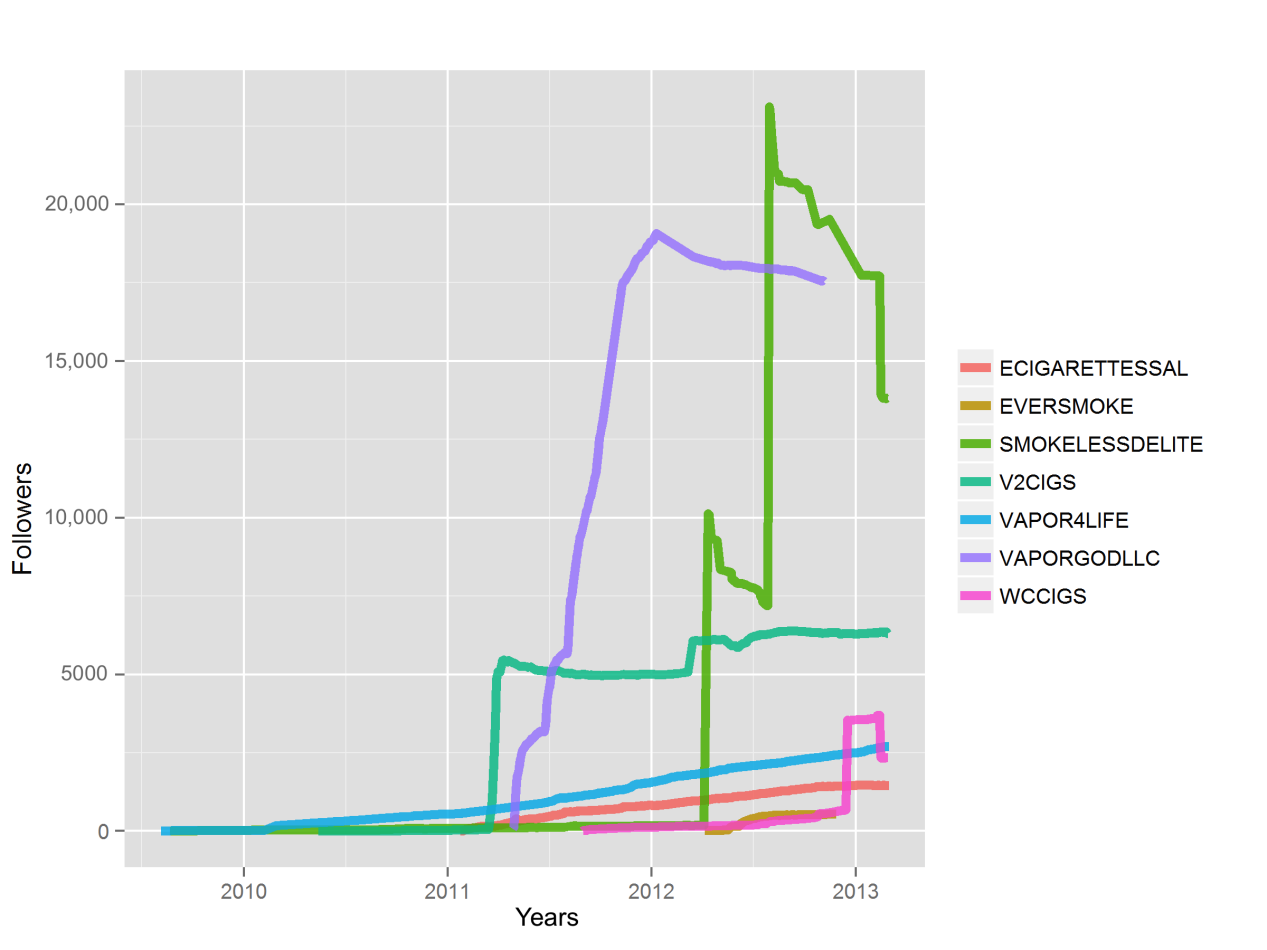
Follower counts of prolific e-cigarette advertising Twitter handles.

For a subset of the most active handles tweeting about e-cigarettes, we explored additional metadata for some insights into how to characterize these users. Specifically, we examined the number of followers accrued over time for the 7 most active tweeters (Figure 2). Amassing a large number of followers in a short time frame, which occurred for certain users, may be suggestive of a marketer or spammer who purchased a list of Twitter handles as followers compared to a legitimate brand or business that accrues followers steadily over time. Indeed, further exploration of historic activities on Twitter during the time in which tweets about e-cigarettes spiked to the highest levels (November 2011-February 2012) revealed that a large number of spam accounts with handles comprising random characters began tweeting around this time and were shut down by Twitter in early 2012.

We coded places where users mentioned using their e-cigarettes or seeing others use e-cigarettes. Of the 471 tweets that mentioned a specific place, 39.1% (184/471) mentioned e-cigarette use in class, whereas 12.5% 59/471) mentioned use in a house/room/bed, 12.1% (57/471) mentioned use in school, 8.7% (41/471) mentioned use in public, 5.7% (27/471) mentioned use in a bathroom, and 4.5% (21/471) mentioned use at work. Some tweets were indeed about individuals using e-cigarettes in public places such as schools (eg, “my teacher yells at me everyday for vaping in class...” and “Vaping in the bathroom #whatofit”) and in the convenience of private spaces such as their bedrooms (eg, “I do love being able to smoke in my room again doe! #ecig”). However, most tweets were from people expressing disbelief at others using e-cigarettes in public places (eg, “Is this guy really smoking an electronic cigarette in class? #Yes #wtf”). People also noted that it was odd to see others use e-cigarettes in places where smoking has traditionally been banned (eg, “The guys in my office are smoking electronic cigarettes, its rather strange seeing smoke indoors, in an office, in the daytime”) and were confused about whether using e-cigarettes in public places is allowed (eg, “My professor was just smoking his electronic cigarette in class, is that illegal?” and “High schoolers smoking e-cigarette in my #metro station. @Wmata Is that allowed? #narc #defnothealthy”).

## Discussion

### Principal Results

In summary, we found that Twitter conversations about e-cigarettes have increased dramatically in recent years. This pattern is consistent with the recent rise in e-cigarette advertising expenditures [8] and e-cigarette use among youth [46] and adults [47]. It is not surprising that the majority of tweets appear to be advertising-related because in the early introduction of a new product claims are often made that new products are more cost-effective (eg, claims that e-cigarettes are cheaper than tobacco cigarettes and nicotine replacement therapy) [48]. Furthermore, because anyone can create a Twitter account and start posting content, e-cigarette vendors can freely “advertise” their products via tweets to the more than 316 million monthly active users with no advertising costs per se. This also means that anyone—whether a legitimate brand, an online-only vendor, an affiliate marketer, or an e-cigarette user/enthusiast—can post content. Indeed, our look at the most active Twitter handles suggests that actual online vendors and potential affiliate marketers are promoting e-cigarettes online, with some potentially using computer programs to generate and post tweet content automatically. This practice is not unique to e-cigarettes as evidenced by the fact that, for years, Twitter has been attempting to identify spam accounts that are generating automated content from computer programs and not actual individuals. This type of “spamming” suggests that there is an online marketplace for e-cigarettes whereby individuals can earn money by driving customers to visit an e-cigarette vendor website. This also means that the online marketplace is more diverse than traditional brick-and-mortar offerings; the top brands mentioned in tweets, except for blu eCigs, are not the leading brands advertised on other media channels (eg, television) or sold in retail stores [8].

The ease of posting user-generated content online and sharing this information across social media platforms such as Twitter suggests that Twitter users may be exposed to more e-cigarette brands and online vendors than non-Twitter users. Although only approximately 23% of the US adult population is on Twitter, use rates are highest among young adults aged 18 to 29 years [17] and have been increasing among youth in recent years [17,49]. Just because youth are on Twitter does not necessarily mean they are exposed to these tweets. One would have to follow these Twitter accounts or be exposed to the tweets through their social network (ie, followers or those they are following) or via searches. This study was not designed to examine audiences that may have been exposed to these e-cigarette tweets; however, recent studies suggest that e-cigarette users learn about e-cigarettes from sources such as the Internet. Therefore, monitoring how e-cigarettes are discussed on social media platforms such as Twitter is important, especially if frequent tweets about e-cigarettes from multiple Twitter handles may give consumers the false perception that e-cigarettes are readily available and use is more common than it really is. Previous research has shown that tactics of fostering a false sense of consensus have been used to oppose passage of e-cigarette policies [28]. We also know from the literature that youth overestimate the prevalence of youth smoking, so exposure to frequent tweets about e-cigarettes and visual cues of people vaping their e-cigarettes via photos may influence their interest in and perceived social norms about e-cigarette use [50].

Our analysis of patterns of tweets among the most active Twitter users also revealed important patterns (eg, accounts gaining many followers during a short window of time) that suggest some of the most active accounts may be engaging in practices such as purchasing followers. Previous research has shown that abnormal patterns in posting behavior (eg, bursts of posts in a short period of time) are indicative of spamming or fake consumers [51]. In this context, a burst of followers for a particular e-cigarette-related Twitter handle likely suggests spamming. Additional work is needed in this area because distinguishing legitimate e-cigarette companies/vendors from spammers/affiliate marketers will help prioritize and inform future regulatory efforts.

Our analysis of nonadvertising tweets indicates that organic conversations about e-cigarettes are occurring online that can provide insights into consumer use behaviors. Interestingly, we find posts about e-cigarettes being used in public spaces, with class being the top mention. Although some of these tweets indicate that students (and staff in some cases) were using e-cigarettes in class, the majority of tweets were from nonusers who expressed surprise or disdain at seeing others using e-cigarettes in public and were confused as to whether this is allowed. We do not know from the tweets whether these were youth or young adults tweeting about a high school class or a college class, and future studies should examine ways to determine the demographics of tweeters. Regardless, these results suggest that youth and young adults may be exposed to e-cigarette use in their everyday lives, and the lack of action by staff may give them a sense that e-cigarette use is permissible and not as harmful as cigarettes. This is a concern among public health professionals and increasingly local governments have introduced [52] and passed [53] policies banning the use of e-cigarettes in public places and in schools (eg, [54]).

### Limitations

This study has several limitations. Although we attempted to identify the entire population of tweets about e-cigarettes using a comprehensive search strategy, we may have missed some relevant tweets. Additionally, we attempted to characterize the most active Twitter handles, but our analysis was based largely on posted information in their profile, which may not reflect who they truly are or their motivations for tweeting e-cigarette content. This is a general challenge in conducting Twitter research and more advanced computational methods are needed to mine the profile descriptions and tweet content of any given Twitter handle to determine the individuals’ demographic characteristics. Additionally, we do not know the extent to which Twitter users were exposed to e-cigarette tweets. Finally, the data presented in this study may have limited implications today given likely changes in the e-cigarette marketplace, brands’ advertising strategies, e-cigarette policies, and consumer behaviors.

### Comparison to Prior Work

This study builds on prior work assessing e-cigarette marketing on Twitter (eg [29]). A key strength is that we analyzed data across multiple years to characterize the emerging trend of e-cigarette conversations on Twitter. We used a data source with access to the full Twitter sample and historical tweets. In contrast, most studies using Twitter data have examined substantially shorter time periods (eg, months) using freely accessible data from the Twitter API, which only provides 1% of all tweets. The Twitter data and analyses presented here also helped us to understand a key period (2008-2013) for e-cigarette marketing when these devices began to increase in popularity. This study also identifies locations where e-cigarettes are being used and suggests the need for better enforcement of policies restricting e-cigarette use in public places such as schools.

### Conclusions

In conclusion, Twitter may provide valuable insights into how information about new products, such as e-cigarettes, are disseminated via social media. Our results suggest that Twitter is being used to promote e-cigarettes and that the online marketplace is more diverse than the offline brick-and-mortar product offerings and advertising strategies. Monitoring and regulating these entities online will be challenging given how easily anyone can set up multiple social media accounts with no verification of their identity before they advertise their products to consumers worldwide. Therefore, it will be important to not only examine the content of e-cigarette advertising tweets, but other characteristics of marketers, such as frequency of tweeting behavior and patterns of acquiring followers, to identify entities that state and federal governments may need to monitor and regulate. For example, enforcing advertising restrictions may have different implications for an actual e-cigarette brand company with brick-and-mortar presence versus an individual who is an affiliate marketer for an online e-cigarette retailer. Our results also highlight that e-cigarettes are being used in public places such as schools and underscore the need for education and enforcement of policies banning e-cigarette use in public places. In summary, Twitter data can provide new insights on a rapidly evolving public health phenomenon such as e-cigarettes to help inform future research, regulations, surveillance, and enforcement efforts.

## Abbreviations

API: application program interface
FDA: Food and Drug Administration
